# Signal-to-Noise Enhancement of a Nanospring Redox-Based Sensor by Lock-in Amplification

**DOI:** 10.3390/s150613110

**Published:** 2015-06-04

**Authors:** Pavel V. Bakharev, David N. McIlroy

**Affiliations:** Department of Physics, University of Idaho, Moscow, ID 83844, USA; E-Mail: bakharevpavel@gmail.com

**Keywords:** MOS, gas sensor, ZnO, lock-in amplifier

## Abstract

A significant improvement of the response characteristics of a redox chemical gas sensor (chemiresistor) constructed with a single ZnO coated silica nanospring has been achieved with the technique of lock-in signal amplification. The comparison of DC and analog lock-in amplifier (LIA) AC measurements of the electrical sensor response to toluene vapor, at the ppm level, has been conducted. When operated in the DC detection mode, the sensor exhibits a relatively high sensitivity to the analyte vapor, as well as a low detection limit at the 10 ppm level. However, at 10 ppm the signal-to-noise ratio is 5 dB, which is less than desirable. When operated in the analog LIA mode, the signal-to-noise ratio at 10 ppm increases by 30 dB and extends the detection limit to the ppb range.

## 1. Introduction

Gas sensors, or chemiresistors, play a critical role in gas and chemical production and high fuel-efficient combustion engines. In addition, they can be used for environmental monitoring [1,2] and to detect hazardous materials, such as explosives vapors [3,4,5,6,7,8,9]. Redox-based sensors, or artificial noses, convert chemical information specific to the analyte into analytical electrical signals [4,5,6,7,8,9,10,11,12,13,14,15,16,17,18]. Hence, they are also excellent scientific tools to analyze molecular interactions at surfaces, be it physisorption or chemisorption. The use of metal oxide nanocrystalline thin films, as well as other more complex nano-morphologies, in the capacity of gas sensitive layers in chemiresistors is well documented [4,5,6,7,8,9,10,11,12,13,14,15,16,17,18]. For metal oxides, the sensing mechanism of the sensors (chemiresistors) is attributed to the depletion, or repletion, of the oxygen at a metal oxide semiconductor (MOS) surface. Consequently, this impacts the depth of the surface depletion layer, thereby producing significant swings in the electrical resistance of a chemiresistor. These studies have demonstrated that a sensor is more responsive to small changes in surface stoichiometry (oxidation state of the metal) if the thickness of the thin film, or the size of the nanostructures, of the MOS layer are comparable to the width of the intrinsic surface depletion layer [4,5,6,7,8,9,10,11,12,13,14,15,16,17,18].

Gas sensor systems should be able to promptly and reliably identify (“detect” and “recognize”) the chemical compound, or compounds, of interest under ambient conditions. One approach to achieve this is to construct a sensor (receptor) array from which a multidimensional electrical response pattern, *i.e.*, a recognition pattern, can be assigned to the compounds. Herein, we present a signal processing methodology using lock-in amplification to increase the signal-to-noise ratio and the absolute detection limit of a single ZnO coated silica nanospring redox-based gas sensor.

## 2. Device Fabrication

The redox-based gas sensor begins with the growth of a mat of silica nanosprings. The details can be found in previous publications by McIlroy *et al.* [19] and Wang *et al.* [20]. The nanosprings are subsequently coated with a ZnO gas sensitive layer by atomic layer deposition (ALD) [21,22]. Zinc oxide ALD coating of silica nanosprings has demonstrated a several advantageous characteristics compared to other deposition methods, such as chemical vapor deposition (CVD), as well as self-assembled ZnO nanowire mat [16,17]. These advantages include, but are not limited to, the ability to precisely control coating thickness of the gas sensitive layer, the size of the ZnO nanocrystals, and the ability to uniformly coat complex 3D structures like nanosprings. ZnO ALD of insulating SiO_2_ nanosprings was conducted in a tube furnace maintained at 175 °C with diethylzinc (DEZn) and deionized water (H_2_O) as the sources of zinc and oxygen, respectively. During this process, 1 Torr background pressure was maintained in the reaction chamber by a continuous flow (6 sccm) of Ar. An ALD cycle started with a DEZn pulse of 150 ms. This pulse was followed by a 10 s pressurizing with Ar (up to 2.5 Torr) and an 8 s pump and an Ar purge. At the next stage, there was a water pulse of 300 ms, followed by another 20 s pump and an Ar purge. A usual ALD process consisted of 150 cycles at an average ZnO thickness of ~70 nm.

**Figure 1:**
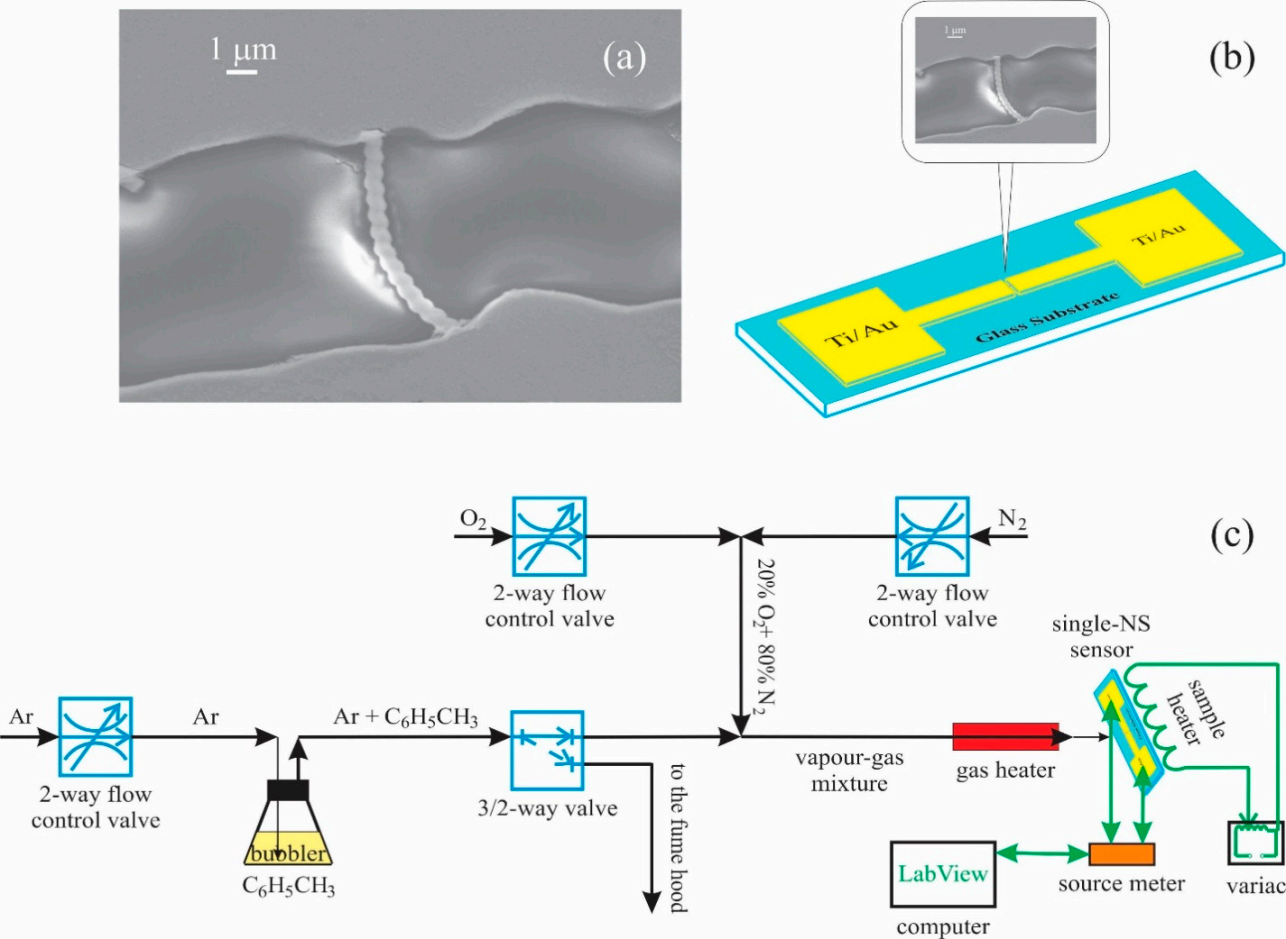
(**a**) SEM image of a single ZnO coated nanospring sensor. Schematics (**b**) of a single ZnO coated nanospring device and (**c**) of the two electrode measurement system used to acquire electrical response of the sensor.

**Figure 2:**
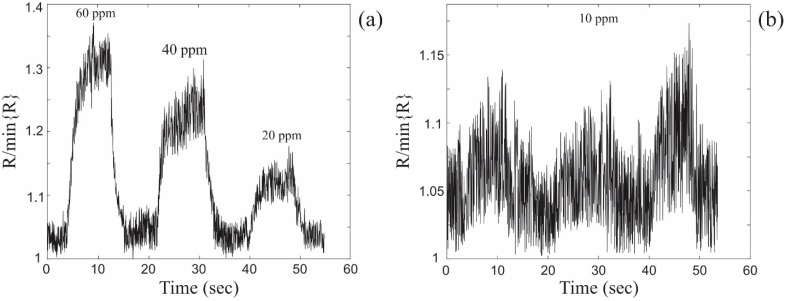
DC electrical response of a single ZnO coated nanospring sensor. The relative changes in resistance upon exposure to toluene vapor at (**a**) 60 ppm, 40 ppm, 20 ppm and (**b**) 10 ppm.

Two terminal single ZnO coated nanospring devices (Figure 1a and schematically illustrated in Figure 2b) were fabricated by suspending the ZnO coated silica nanosprings in isopropyl alcohol (IPA) and transferring the nanospring-alcohol solution onto 25 mm × 25 mm microscopic glass slides and allowing the IPA to evaporate. The electrical contacts (50 nm of Ti layer followed by 150 nm of Au layer) with 10 μm spacing were applied to the glass substrate using standard photolithography and lift-off techniques. SEM image of an individual ZnO coated nanospring spanning Ti–Au electrical contacts is displayed in Figure 1a. Any additional nanosprings lying between the electrodes were removed under an optical microscope using a microprobe needle, thereby ensuring that the electrical response is of a single nanospring.

## 3. Electrical Characterization

The single nanospring device was mounted to a heating plate to enable temperature regulation. Two electrical probes were used to make electrical contact to the Ti–Au pads. The electrical measurements were acquired using a Kiethley 2400 source-sense meter interfaced to a computer via Labview-operated data acquisition software for real time resistance measurements. The single nanospring chemiresistor was placed in a chamber through which a continuous flow of synthetic air (20% O_2_ and 80% N_2_) was maintained at all times. Sequential pulses of toluene vapor were generated by the constant flow of Ar gas through a bubbler of liquid toluene and a solenoid valve placed downstream of the bubbler (see Figure 1c). Prior to exposure to toluene vapor, the sensors were allowed to reach a steady state resistance in the synthetic air at atmospheric pressure.

The contribution of the glass substrate to the total electrical conductivity of the device was examined by comparing the I-V characteristics of the Ti–Au terminals with and without the presence of a ZnO coated nanospring [5]. In the absence of a nanospring, at a temperature of 300 °C, an open circuit with a nominal current (orders of magnitude less than with a nanospring present) was observed, where the nominal current is attributed to residual carbon on the glass surface. Moreover, unlike the single nanospring device, the electrodes devoid of ZnO coated nanospring did not respond to the analyte (toluene) vapor.

The DC electrical responses of the single ZnO coated nanospring chemiresistor operated at 310 °C to toluene partial vapor pressures of 60 ppm, 40 ppm, 20 ppm, 10 ppm are shown in Figure 2. The chemiresistor exhibits good linearity of the calibration graph (response vs analyte concentration) with a slope of 18% response change per 40 ppm toluene partial pressure change.

The overall noise in the electrical signal illustrated in Figure 2 has multiple sources and, in general, can be subdivided into intrinsic and extrinsic noise sources.

There are many sources of intrinsic noise, each having unique characteristics. For DC measurements conducted for the single ZnO coated nanospring device, the overall noise is comprised of three main constituents, which are thermal noise (Johnson-Nyquist noise), shot noise, and flicker (1/*f*) noise [23,24]. Thermal noise is the electronic noise generated by the thermal equilibrium fluctuations of charge carriers. This noise type does not depend on applied voltage. It appears as white noise with power density spectrum given by,

(1) $$S_{\mathrm{Th}}=4k_{\text{B}}T_{\mathrm{NS}}R$$

where *k*_B_ is the Boltzmann constant, *T*_NS_ is the absolute temperature of the sensor, and *R* is the resistance of the individual ZnO coated nanospring. Heating of the ZnO thermally activates surface sites (such as oxygen vacancies), which subsequently chemisorb atmospheric oxygen. This, in turn, increases the sensor resistance *R*. Hence, according to Equation (1), at elevated temperatures the thermal noise prevails over other intrinsic noise sources.

With that said, shot noise and flicker (1/*f*) noise can become dominant sources of noise at low temperatures with diminished thermal noise [5]. Shot noise is a white noise source associated with the discreteness of the electric charge with a power density spectrum, *S*_Shot_, given by,

(2) $$S_{\mathrm{Shot}}=2eI$$

where *e* is the electron charge and *I* is the average current in the device. Shot noise can be observed in electronic devices with internal potential barriers, such as potential barriers formed at crystal grain interfaces and Schottky contacts.

Two major theories have been developed to explain the physical origin of flicker (1/*f*) noise in MOS devices, namely, the number fluctuation theory (NFT) [25,26,27] originally proposed by McWhorter [28] and the bulk mobility fluctuation theory (MFT) based on Hooge’s model [29]. According to the NFT, flicker noise arises from random trapping and detrapping of carriers at defects near the MOS surface and near the MOS/insulator interface (ZnO/SiO_2_ interface in this study). Hence, this type of noise can be observed mainly in nanoscale MOS devices with a high surface-to-volume ratio, which is the case for ZnO coated nanosprings. The mobility fluctuation theory attributes the 1/*f* noise to fluctuations in bulk mobility caused by phonon scattering. According to Hooge's empirical relation, the power density spectrum *S*_Fl_ can be expressed as

(3) $$S_{\mathrm{Fl}}=\alpha_{\text{H}}\frac{V^{2}}{N\;f}$$

where *N* is the total number of carriers in the semiconductor, *V* is the applied bias voltage, and α_H_, known as Hooge’s parameter, is an empirical constant used as the measure of the noise magnitude [29].

Extrinsic noise sources include electromagnetic fields that couple into sensitive circuit (radiative coupling, capacitive coupling, inductive coupling, and conductive coupling), mechanical vibrations that trigger piezoelectric materials such as ZnO thin films [30,31] to generate unintended AC electrical signals, or in the present case, noise from random fluctuations in concentrations of flowing gases and vapors.

**Figure 3:**
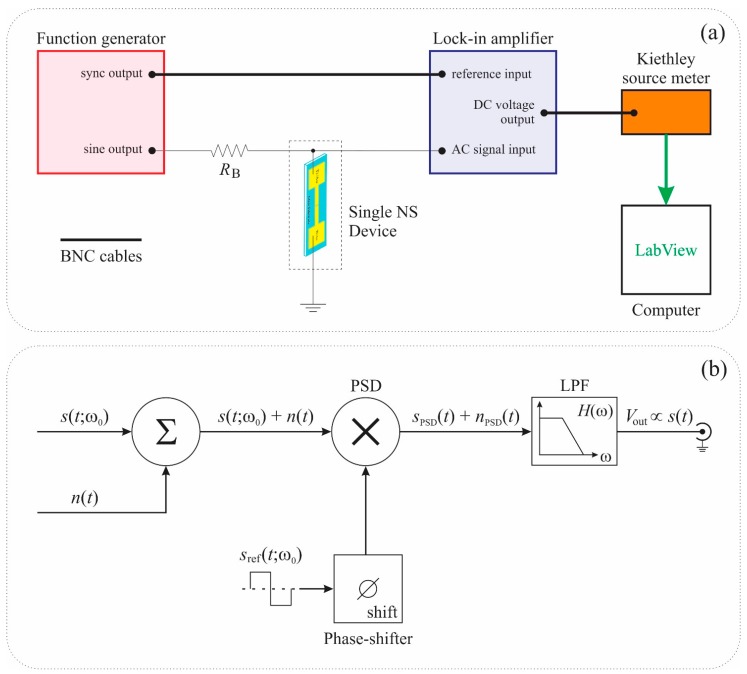
(**a**) Schematic of the lock-in based experimental set-up utilized for toluene vapor detection; (**b**) Layout of signal pathway through an analog lock-in amplifier (LIA). The symbols Σ and × represent summing and mixing (multiplying) stages, respectively. Phase-Sensitive Detector (PSD) and low-pass filter (LPF) are a multiplier, called the phase sensitive detector, and a low-pass filter with transfer function *H*(ω), respectively.

In order to significantly increase the signal-to-noise ratio (SNR) and subsequently extend the lower detection limit of a ZnO-coated silica nanospring chemiresistor/sensor, the analog lock-in amplifier (LIA) technique has been integrated into the signal processing of the sensor signal. The inclusion of LIA changes the operating mode of the sensor from DC to AC through the introduction of a modulated input signal generated by a function generator. The AC electrical response measurements were carried out using a Stanford Research Systems SR510 analog LIA. The measurement scheme of the lock-in based experimental set-up used to detect small amplitude modulations in AC signals of the single ZnO coated nanospring chemiresistor is shown in Figure 3a.

A layout of noisy signal pathway through an analog LIA is illustrated in Figure 3b. An input time-dependent signal *s*(*t*; ω_0_) in the presence of noise *n*(*t*) is multiplied with the reference analog LIA waveform *s*_ref_(*t*; ω_0_) at the multiplier, also known as the Phase-Sensitive Detector (PSD). PSD generates a signal, the DC component of which is proportional to the amplitude of the AC input signal *s*(*t*) and depends on the phase difference between the input signal and the reference signal. The DC component in the PSD output signal can be extracted by means of the low-pass filter (LPF) with the transfer function *H*(ω) and the characteristic band-width $\Delta \omega_{\mathrm{LPF}}$. The lock-in quality factor Q can be expressed as,

(4) $$Q=\frac{\omega_{0}}{\Delta \omega_{\text{LPF}}}$$

The SNR can be defined as the power ratio of a signal to the background noise:

(5) $$\mathrm{SNR}=\frac{\bar{P_{s}(t)}}{\bar{P_{n}(t)}}$$

where $\bar{P_{s}(t)}$ and $P_{n}(t)$ are the average spectral powers of the signal and the background noise, respectively. In decibel scale, the SNR is given as,

(6) $$\mathrm{SN}{\text{R}}_{\mathrm{dB}}=10\log_{10}(\mathrm{SNR})$$

The mean square value of the noise amplitude should be used to estimate the experimental SNR, since the background noise is a random signal with zero mean value.

The noise obtained from the DC measurements, as well as the noise at the LIA input *n*(*t*) with power spectral density *N*(ω), can be represented as the following limit:

(7) $$n(t)=\underset{\Delta \omega \to 0}{\lim}\sum_{i=-\infty }^{+\infty }C_{i}\sin\left(\omega_{i}t+\theta_{i}\right);\;\;\;\omega_{i}=i\Delta \omega$$

where *C_i_* is the Fourier coefficient. Upon the transition through the LIA with the low-pass filter (LPF) transfer function *H*(ω), the mean-square value of the output noise is,

(8) $$\bar{n_{\mathrm{out}}^{2}(t)}=\frac{1}{2\pi }\int_{-\infty }^{+\infty }N_{\mathrm{PSD}}(\omega ){\left|H(\omega )\right|}^{2}d\omega$$

where *N*_PSD_(ω) is the power spectral density of the noise upon passage through the PSD. The transfer function, *H*(ω), of the *RC* low-pass filter (LPF) is,

(9) $$H\left(\omega \right)=\frac{1}{1+i\omega \tau_{RC}}$$

where $\tau_{RC}$ is a characteristic time constant of the LPF. Note, the goal of this work is not to identify the dominant noise mode of the chemiresistor, but to increase the SNR. Identification of the degree to which the different types of noise contribute to the signal will be the subject of a future study.

**Figure 4:**
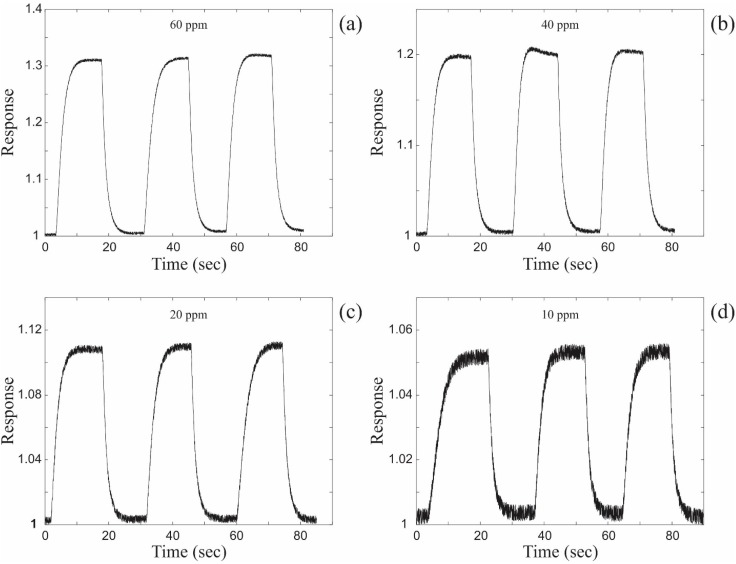
The electrical response of a single ZnO coated nanospring device measured at lock-in amplifier (LIA) output upon exposure to toluene vapor at (**a**) 60 ppm; (**b**) 40 ppm; (**c**) 20 ppm and (**d**) 10 ppm.

The electrical response of a single ZnO coated nanospring chemiresistor measured at LIA output to toluene vapor pulses at 60 ppm, 40 ppm, 20 ppm and 10 ppm is displayed in Figure 4. The LIA chemiresistor signals in Figure 4 are superior to the DC signals in Figure 2. The contribution of noise to the signal of the chemiresistor in LIA mode is practically negligible down to 20 ppm. At 10 ppm of toluene the signal of the chemiresistor operated in the DC mode is not trustworthy. By contrast, when operated in LIA mode, the chemiresistor signal at 10 ppm is as reliable as that at 60 ppm. Note, there is no loss of information with regards to the amplitude of the signal when operated in the LIA mode. The utilization of the LIA technique significantly improves not only the electrical response characteristics of a single ZnO coated nanospring sensor (receptor), but also considerably expands the recognition capabilities of the gas sensor array (electronic nose) in the frames of linear discrimination analysis (LDA), independent component analysis (ICA), principal component analysis (PCA) and other multiple odor recognition methods [9,11,32,33,34]. The repeatability of the signal profile and intensity will greatly increase the reliability of LDA-based detection.

**Figure 5:**
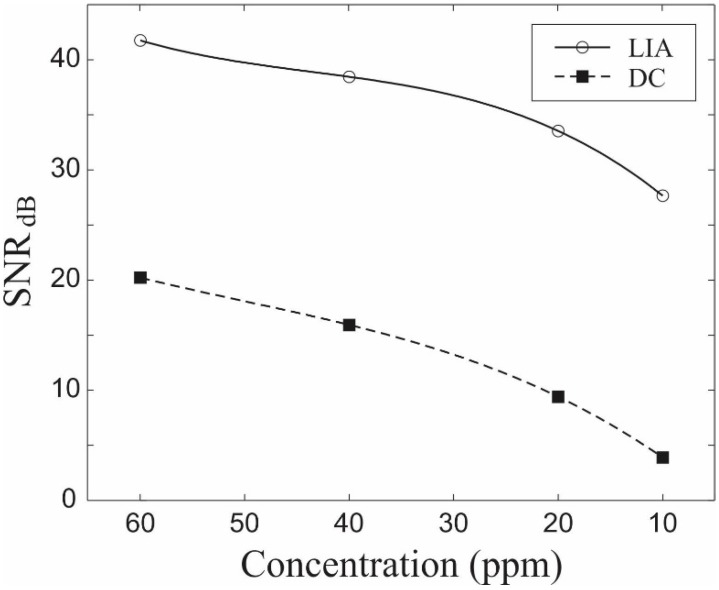
Signal-to-noise ratios (SNRs) in decibel scale of a single ZnO coated nanospring sensor obtained by utilizing the DC and the analog lock-in amplifier (LIA) modes of detection.

A summary SNR of the chemiresistor operated in DC and LIA modes is presented in Figure 5, which clearly demonstrates the significant increase in the SNR at all concentrations upon signal passage through the LIA. This effect is attributed to the fact that only the low-frequency components of the noise power spectral density, *N*_PSD_(ω), contribute to the LIA output noise within its equivalent noise bandwidth (ENBW) $\Delta \omega_{\mathrm{LPF}}$. The ENBW, $\Delta \omega_{\mathrm{LPF}}$, of the *RC* LPF is approximately equal to $1/\tau_{\mathrm{RC}}$. For practical purposes of gas sensors, real-time response measurements are required, so the integration timescale of LIA should be low enough to facilitate relatively short detection response times. Hence, the characteristic time constant, $1/\tau_{\mathrm{RC}}$, of the *RC* LPF was set to be 50 ms since it is much shorter than the sensor response time [5,6] and simultaneously very long compared to the period, $T_{0}$, of the reference signal ($T_{0}=10\;\mathrm{ms}$). Comparing the SNRs of the DC and LIA measurements performed for a single ZnO coated silica nanospring, the utilization of the conventional analog LIA leads to the sensor resolution reduction by a factor of 20.

Hence, the analog LIA measurements of small AC modulation signals generated by the redox and the analyte oxidation processes at the ZnO surface [5,6,7,8,9] enables a detection limit of the sensor to be reduced from tens of ppm (for DC measurements) to tens of ppb range under equivalent experimental conditions.

## 4. Conclusions

A new nanomaterials-based chemical sensor (chemiresistor) has been developed that has excellent sensitivity and SNR. The chemiresistor has been constructed with a single ZnO coated silica nanospring. The gas sensor response to the analyte (toluene) vapor is attributed to its catalytic oxidation of the analyte at the ZnO surface, hence creating an oxygen deficient surface of ZnO, and self-refreshing through dissociative chemisorption of oxygen (redox process). DC mode chemiresistor responses to pulses of toluene vapor at the ppm concentration levels demonstrate high sensitivity and the detection limit of 10 ppm of the single ZnO coated nanospring chemiresistor. By comparison, when the nanospring chemiresistor is operating in the analog lock-in amplifier mode a superior SNR is achieved and the detection limit is extended to tens of ppb. In conclusion, it has been shown that the operation of a chemiresistor operated in the analog LIA mode significantly increases the SNR, improves sensitivity and considerably lowers the detection limit. The LIA operating mode can be used with essentially any chemiresistor, or other types of chemical sensors, especially when employing LDA and ICA signal recognition.
